# Intestinal Necrosis due to Giant Ovarian Cyst: A Case Report

**DOI:** 10.1155/2013/831087

**Published:** 2013-12-16

**Authors:** Ali Duran, Fulay Yilmaz Duran, Fevzi Cengiz, Ozgur Duran

**Affiliations:** ^1^Department of General Surgery, Izmir Bozyaka Educational and Research Hospital, 35140 Izmir, Turkey; ^2^Department of Anesthesiology and Reanimation, Izmir Bozyaka Educational and Research Hospital, 35140 Izmir, Turkey; ^3^Department of Emergency Medicine, Izmir Bozyaka Educational and Research Hospital, 35140 Izmir, Turkey

## Abstract

Intestinal pathologies due to ovarian cyst are observed rarely. Although a limited number of cases in neonatal and adolescent periods have been observed, no adult case has been reported in the literature. Two mechanisms are involved in intestinal complications due to ovarian cysts: torsion due to adhesion or compression of giant ovarian mass with a diameter of 9-10 cm. We report here a terminal ileum necrosis case due to compression by an ovarian cyst with 11 × 10 × 7 cm size in an 81-year-old woman.

## 1. Introduction

Although ovarian cysts are commonly observed in women, those with sizes huge enough to fill in the pelvic cavity are rare. These giant cysts may not cause a symptom until reaching a certain size. On the other hand, depending on the localization, size, and presence of compression, they may cause acute abdomen (bleeding, rupture, and obstruction) leading to such symptoms as abdominal pain, nausea, vomiting, and constipation. Gynecologic disorders presenting with acute abdominopelvic pain incidence have been reported to comprise 1.5% of office-based visits and 5% of emergency department admissions [1, 2]. Radiologic tools are essential for diagnosis.

Intestinal pathologies due to ovarian cyst are observed rarely. Although limited number of cases in neonatal and adolescent periods has been observed, no adult case has been reported in the literature. We report here a case of terminal ileum necrosis due to the compression by a giant ovarian cyst who underwent urgent surgery.

## 2. Case Report

**Figure 1:**
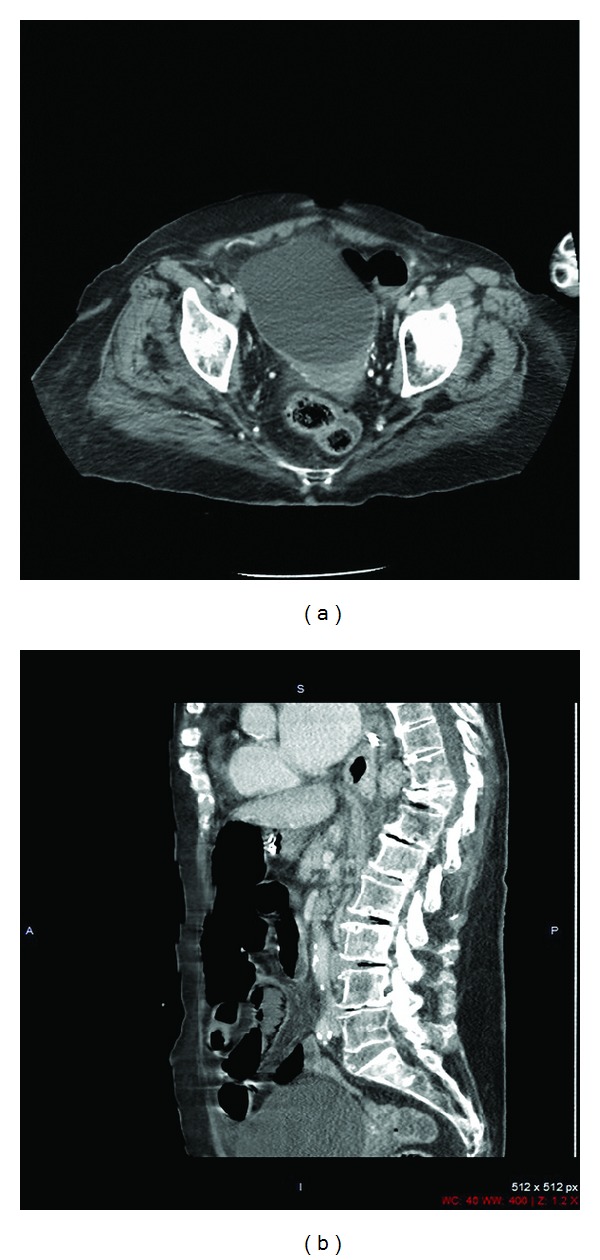
A 10 × 10 cm cystic mass on the right ovary and jejunal and ileal segments observed above, below, and behind the mass detected by abdominal computer tomography.

**Figure 2:**
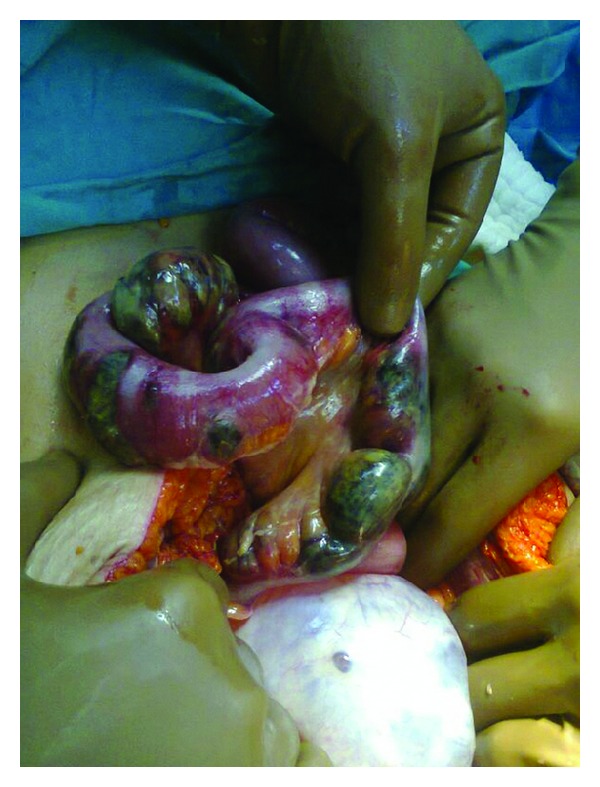
Right ovarian cystic mass and obstructed and necrotic terminal ileum due to this cystic mass.

An 81-year-old woman admitted to emergency department due to abdominal pain, nausea, and vomiting which have been lasting for 4 days. The patient's general condition was serious on admission. Her history revealed no health problem except for a right adnexal cyst. Distended abdomen, hypoactive bowel sounds, and abdominal guarding and rebound tenderness were found on physical exam. Laboratory workup revealed WBC: 26200 mm^3^, urea: 123 mg/dL, creatine: 0,97 mg/dL, glucose: 346 mg/dL, and amylase: 256 U/L. Fluid levels were observed in upright abdominal X-ray and dilated intestinal loops, and a cystic mass were detected in abdominal ultrasound. Abdominal computer tomography revealed air-fluid levels in jejunal and ileal segments, minimal fluid between loops and a 10 × 10 cm cystic mass on the right ovary (Figures 1(a) and 1(b)). The patient underwent emergency surgery. It was noted, in the course of surgery, that the blood circulation of terminal ileum segments that were obstructed and compressed by the right ovarian cystic mass was blocked (Figure 2). The terminal ileum and caecum were resected by right hemicolectomy, and side-to-side anastomosis was performed. The patient died 6 h after the surgery due to cardiopulmonary arrest. Macroscopic evaluation of the specimen revealed that the ovarian cyst had 11 × 10 × 7 cm dimensions and 382 g weight, and the width of the intestinal wall was reduced to 0.1 cm at necrotic sites. Benign cystic adenoma, intestinal ischemia, and necrosis were reported in histopathologic evaluation.

## 3. Discussion

Simple ovarian cysts are the most common nonneoplastic adnexal masses among women of reproductive age [3]. It is now known that the cyst-producing capabilities of the ovary do not cease with menopause [4]. Cysts with a diameter of greater than 10 cm may cause abdominal pain, vaginal bleeding, and swelling. These cysts have malignancy risk, and morphological changes and increased serum CA 125 levels during follow-up are indications for surgery [4]. The incidence for postmenopausal asymptomatic ovarian cyst varies between 3% and 18% [4]. The improvement in image quality of ultrasound technology facilitates the diagnosis and follow-up of the adnexal masses in postmenopausal women. The patient described in the present report had an adnexal mass which was followed up for a long period of time. During the follow-up the patient did not express any symptom due to this mass, except for occasional abdominal pains. The relevant literature reveals rare intestinal obstruction and perforation cases due to ovarian cyst in the neonate, but not in adults. The incidence of ovarian cysts complicated with intestinal obstruction in neonatal period is 3% [5]. The symptoms include abdominal pain and swelling. We detected nonspecific symptoms such as abdominal pain, swelling, nausea, and vomiting in our case, but such symptoms specific to women as vaginal bleeding were negative.

Acute mechanical intestinal obstructions (AMIOs) are observed commonly in emergency surgery practice. Localizations of intestinal obstructions that prevent distal transfer of gastrointestinal content are classified into three as follows: intestinal lumen, intestinal wall, or extraintestinal localization. AMIOs occur generally due to adhesions as a complication of past abdominal operations, as well as invagination, polypoid tumor, gallstone, bezoar formation, foreign body, intestinal parasites, trauma, tumor, abdominal inflammation, external-internal herniation, volvulus, stricture due to inflammatory bowel disease, submucosal hematoma due to long-term anticoagulant use. None of these etiologies was present in our case. We detected oliguria and azotemia due to hypovolemia caused by prolonged AMIO and vomiting, as well as reduced central venous pressure and cardiac output, hypotension, and hypovolemic shock which lead to irreversible dehydration and hemoconcentration and deteriorated the patient's general condition [6]. Therefore, we believe that the choice of treatment made by correct and timely diagnosis of the etiology may decrease the mortality and morbidity. Besides the giant ovarian cystic mass, all the radiological workup revealed findings of mechanical intestinal obstruction. Two mechanisms of intestinal obstruction due to an ovarian mass have been proposed: first, the mass may cause torsion due to adhesions that may rarely cause intestinal obstruction, and, second, a giant mass may cause compression [5]. We detected, in our case, obstruction and necrosis of terminal ileum and caecum due to compression of an ovarian giant mass.

In conclusion, intestinal pathologies due to giant ovarian cysts should be considered in women admitted to emergency service with symptoms of acute abdomen.
